# *Porphyromonas gingivalis* outer membrane vesicles prevent the induction of tolerogenic dendritic cells by first trimester trophoblast cells

**DOI:** 10.3389/fimmu.2026.1814664

**Published:** 2026-05-08

**Authors:** Brenda Lara, Ana Schafir, Ailén Fretes, Lourdes Materazzi, Fátima Merech, Daiana Rios, Rosanna Ramhorst, Claudia Pérez Leirós, Daiana Vota, Soledad Gori, Vanesa Hauk

**Affiliations:** Universidad de Buenos Aires – Consejo Nacional de Investigaciones Científicas y Técnicas (National Scientific and Technical Research Council) (CONICET), Instituto de Química Biológica de la Facultad de Ciencias Exactas y Naturales (IQUIBICEN), Buenos Aires, Argentina

**Keywords:** immunomodulation, *P. gingivalis* outer membrane vesicles, periodontal disease, tolerogenic dendritic cells, trophoblast cells

## Abstract

**Introduction:**

Periodontitis is a risk factor for adverse pregnancy outcomes, yet the mechanisms linking *Porphyromonas gingivalis* (Pg) to placental immune dysregulation remain unclear. Outer membrane vesicles (OMV) derived from this pathogen act as systemic virulence mediators, but their impact on maternal–fetal immune tolerance is unknown. Dendritic cells play a central role in integrating trophoblast-derived signals to maintain immune homeostasis during early pregnancy; therefore, we investigated whether PgOMV reprogram trophoblast immunomodulatory function and thereby affect monocyte recruitment and dendritic cell differentiation.

**Methods:**

First-trimester trophoblast cell line was exposed to PgOMV, and conditioned media were used to evaluate monocyte migration and dendritic cell differentiation *in vitro*.

**Results:**

PgOMV priming increased granulocyte–macrophage colony-stimulating factor and C–C motif chemokine ligand 2 expression and enhanced monocyte migration. While Tb CM impaired monocyte-to-dendritic cell differentiation and promoted a tolerogenic phenotype characterized by low CD86 expression, increased interleukin-10 production and a higher IL-10/TNF-α ratio, PgOMV exposure abrogated this regulatory program and restored inflammatory maturation, including responsiveness to lipopolysaccharide.

**Discussion:**

These findings demonstrate that PgOMV reprogram trophoblast secretory activity, disrupt tolerogenic dendritic cell differentiation and provide a mechanistic link between periodontal infection and impaired immune regulation during pregnancy

## Graphical Abstract

**Figure:**
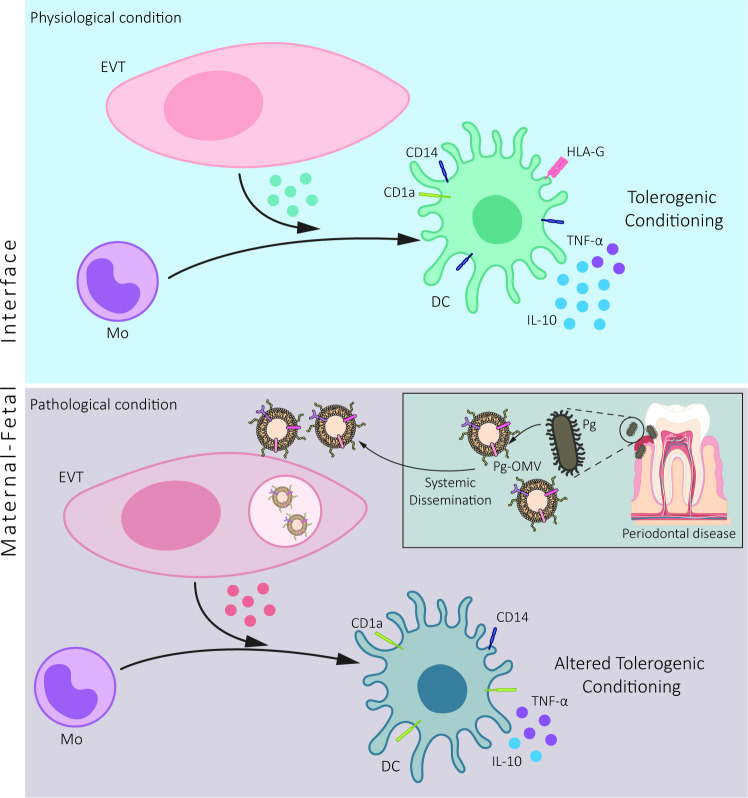
Under physiological conditions, trophoblast cells induce tolerogenic dendritic cells (DCs) from recruited monocytes, promoting maternal–fetal tolerance. In contrast, PgOMVs disrupt this conditioning, leading to a pro-inflammatory DC phenotype.

## Introduction

Periodontitis affects approximately 20-80% of pregnant women globally and is a recognized risk factor for adverse pregnancy outcomes (APOs), including preterm birth, fetal growth restriction (FGR), low birth weight, and preeclampsia (PE) (1–5). Although numerous clinical studies and meta-analyses support this association, the underlying biological mechanisms linking periodontal disease to pregnancy complications remain poorly understood.

Among periodontal pathogens, *Porphyromonas gingivalis* (Pg) is considered a primary etiological agent of periodontitis and has been implicated in systemic inflammatory and vascular conditions, including adverse pregnancy outcomes (6, 7). Pg releases outer membrane vesicles (OMV), nanoscale structures (80–150 nm) enriched in virulence factors that facilitate bacterial interaction with host cells and dissemination to distant tissues (8–10). This dissemination capacity has been proposed to underlie the involvement of PgOMV in the pathogenesis of systemic diseases such as cardiovascular disease, rheumatoid arthritis, and Alzheimer’s disease (11–14). In line with this, we have previously demonstrated that PgOMV are internalized by trophoblast cells, impair trophoblast function, and increase susceptibility to APOs (15).

At the maternal-fetal interface, extravillous trophoblasts (EVTs) are primarily responsible for anchoring the placenta to the uterine wall and remodeling the maternal spiral arteries, processes that are essential for adequate placental perfusion and fetal development (16). Beyond these structural and vascular roles, EVTs also exhibit sentinel and immunomodulatory functions. In fact, trophoblast cells play a central role in establishing and maintaining immune homeostasis, which is essential for successful pregnancy. These cells sense and respond to signals from decidual and immune cells through pattern recognition receptors, including Toll-like receptors (TLRs), allowing them to detect both damage- and pathogen-associated molecular patterns (DAMPs and PAMPs). In addition to their sentinel role, trophoblasts actively modulate immune cell differentiation and function, thereby promoting maternal–fetal tolerance (17–20).

Consistent with this immunomodulatory capacity, we have previously shown that the first-trimester trophoblast cell line Swan-71 induces regulatory and tolerogenic profiles in monocytes and dendritic cells (DCs) (21, 22). In particular, trophoblast-conditioned tolerogenic DCs (Tol-DCs) promote regulatory T cell differentiation and suppress inflammatory responses (22). The clinical relevance of these regulatory DC populations is underscored by altered frequencies of Tol-DCs reported in first-trimester reproductive complications such as recurrent pregnancy loss, suggesting a critical role for regulatory DCs in pregnancy maintenance (23–27). Consistently, disruption of trophoblast–DC interactions can lead to pregnancy complications including PE (28–30).

Although we previously reported that trophoblast cells primed with PgOMV exhibit enhanced neutrophil chemoattraction and a loss of their anti-inflammatory properties (31), the impact of PgOMV on other myeloid immune cells at the early maternal-fetal interface, particularly DCs, remains unexplored. In particular, whether PgOMV can modulate trophoblast immune signaling in a way that influences monocyte recruitment and dendritic cell differentiation at the maternal–fetal interface remains unknown.

Based on these observations, we hypothesized that PgOMV disrupt trophoblast-mediated conditioning of monocyte-derived DCs, preventing the establishment of immune tolerance during early pregnancy. Therefore, the aim of this study was to investigate whether PgOMV alters the ability of first-trimester trophoblast cells to induce tolerogenic monocyte/DC profiles using *in vitro* co-culture models.

## Materials and methods

### Reagents

Endotoxin-free reagents and plastic materials were used in all experiments. RPMI-1640, Dulbecco’s modified Eagle’s medium (DMEM), penicillin/streptomycin, and phosphate-buffered saline (PBS) were obtained from Gibco (Grand Island, NY, USA). Fetal bovine serum (FBS) was sourced from Internegocios (FRA, Buenos Aires, Argentina). Twenty-four-well flat-bottom polystyrene plates were acquired from Jet-biofil (Guangzhou, China), while 96-well U-bottom plates and half-area 96-well ELISA plates were procured from Greiner Bio One (Kremsmünster, Austria). Ficoll-Paque PLUS and Percoll were supplied by Cytiva (Marlborough, MA, USA). Recombinant human IL-4 and recombinant human granulocyte-macrophage colony-stimulating factor (GM-CSF) were obtained from Miltenyi Biotec (Bergisch Gladbach, Germany). Lipopolysaccharide (LPS, *Escherichia coli* O127:B8) were purchased from Sigma-Aldrich (St. Louis, MO, USA).

### *Porphyromonas gingivalis* outer membrane vesicles isolation

OMV were isolated as previously described (15, 31, 32). Briefly, *Porphyromonas gingivalis* strain ATCC 33277 was cultured anaerobically at 37°C in ATCC Medium 2722 (Tryptic Soy Broth 30.0 g/L and Yeast Extract 5.0 g/L) supplemented with 5% L-cysteine, 5 mg/L hemin, and 1 mg/L vitamin K1 (Sigma-Aldrich, St. Louis, MO, USA). Bacteria were cultured in anaerobic conditions using Anaerocult^®^ A gas-generating systems (Merck) in sealed anaerobic jars for a total of 5 days until reaching the early stationary phase, defined as OD_600nm_ = 1.0–1.2 under our culture conditions. Bacterial growth was monitored by measuring optical density at 600 nm, and this growth phase was consistently reached between days 4 and 5 of incubation. Bacterial cells were removed by centrifugation at 8,000 × g for 10 minutes at 4°C. Supernatants containing PgOMV were filtered through a 0.22-μm filter (GE Healthcare Life Sciences) and subsequently subjected to ultracentrifugation at 100,000 × g for 70 minutes at 4°C. The resulting pellet was resuspended in PBS, sterilized by filtration through a 0.22 μm filter, and stored at -80°C until use. The protein concentration of OMV used for the experiments was determined using the Pierce bicinchoninic acid (BCA) protein assay kit. OMV preparations were routinely checked for bacterial contamination by plating on blood agar plates and incubating anaerobically for 7 days. Only OMV batches showing no bacterial growth were used for the experiments. Vesicle isolation and characterization were performed following the Minimal Information for Studies of Extracellular Vesicles (MISEV) guidelines. In our previous study (32), PgOMV were characterized by nanoparticle tracking analysis (NTA), transmission electron microscopy (TEM), and proteomic profiling, showing a mean diameter of 168.2 ± 8.7 nm. Based on NTA measurements, 1 μg of OMV-associated protein corresponds to approximately 7.0 × 10^8^ particles.

### Trophoblast cell culture and conditioned media preparation

Human first trimester extravillous trophoblast cell line HTR-8/SVneo was cultured in 24-well plates until reaching 80% confluence using complete medium (DMEM-F12 with 10% FBS, 50 U/ml penicillin, 50 μg/ml streptomycin) as previously described (15, 31, 33) and were used between passage number 6 and 12. To obtain trophoblast-conditioned media for monocyte/DCs cultures, 4 × 10^5^ HTR-8/SVneo cells were cultured in complete DCs media (RPMI-1640 with 10% FBS, 50 U/ml penicillin, and 50 μg/ml streptomycin) for 20 h in the presence of 1 μg/ml PgOMV or vehicle control (PBS). The concentration of 1 μg/mL PgOMV was selected based on previous studies from our group showing that this range induces measurable cellular responses without causing cytotoxic effects (15). Following incubation, the culture supernatants were centrifuged at 2000g for 10 minutes to remove cellular debris. The resulting supernatants, termed trophoblast-conditioned media (Tb CM) and PgOMV-treated trophoblast-conditioned media (PgOMV Tb CM), respectively, were collected and stored at -80°C until used. Experimental conditions were processed in parallel and analyzed using standardized protocols to minimize variability.

### Real Time quantitative PCR

RT-qPCR was performed on RNA extracted from HTR-8/SVneo extravillous trophoblast cells. Total RNA was extracted using TriReagent according to the manufacturer’s instructions. A total of 1μg RNA was reverse-transcribed using MMLV reverse transcriptase, RNAse inhibitor, and oligo (dT) kit, and the cDNA was amplified using FastStart Universal SYBR Green PCR Master Mix (Cat. 4913850001, Roche, Sigma) with specific primers (Macrogen, South Korea, **Table 1**) (15). A Bio-Rad iQ5 RT-PCR system was used and gene expression was quantified relative to the mRNA expression of the endogenous reference gene YWHAZ by comparative Ct method, using *2^-ΔΔCt^* calculation and expressed as fold change to non-treated cells (arbitrary units or A. U.). qPCR reactions showed efficiencies within the acceptable range (90–110%), linear standard curves (R² ≥ 0.98), and single-peak melt curves, indicating specific amplification and absence of primer-dimers.

**Table 1:** Primer sequences.

Gene	Primer forward (5′→3′)	Primer reverse (5′→3′)
CCL-2	CAGCAGCAAGTGTCCCAAAG	GAGTGAGTGTTCAAGTCTTCGG
GM-CSF	TCTCAGAAATGTTTGACCTCCA	GCCCTTGAGCTTGGTGAG
YWHAZ	CAGAGAGAAAATTGAGACGGAGC	GTGACTGATCGACAATCCCTTTC

CCL-2, C–C motif chemokine ligand 2; GM-CSF, Granulocyte–macrophage colony-stimulating factor; YWHAZ, Tyrosine 3-Monooxygenase/Tryptophan 5-Monooxygenase Activation Protein Zeta.

### Blood samples and monocytes isolation

Buffy coats were collected from healthy, non-pregnant female volunteers who had not received any pharmacological treatment for at least 10 days prior to blood donation at Fundación Hemocentro Buenos Aires, after providing written informed consent. The Research and Ethics Committee of the Academia Nacional de Medicina (CABA, Argentina) approved this study. A total of nine independent donors were used across the study, and the number of donors included in each experiment is indicated in the corresponding figure legends.

Peripheral blood mononuclear cells (PBMCs) were isolated from buffy coats by Ficoll-Paque PLUS density gradient centrifugation (1.077 g/mL) as previously described (34–36). Monocytes were purified using a hyper-osmotic Percoll gradient as described in 34. Monocytes were collected from the gradient interface, washed, and resuspended in complete DCs medium. Purity (>85%) and viability (>95%) were confirmed by flow cytometry and trypan blue exclusion, respectively. All experiments were performed independently using different donor monocytes (n is indicated in the legend of each figure).

### Monocyte migration assays

The ability of the extravillous trophoblast cells to recruit monocytes was quantified using a transwell migration assay. Briefly, 2 × 10^5^ monocytes were seeded in 3 µm-transwell systems that were placed in a lower chamber containing Tb CM or PgOMV-pretreated Tb CM (PgOMV Tb CM). After 30 min at 37°C, the number of recruited monocytes was determined via flow cytometry as described in 21. Migrated monocytes were quantified as single measurements per donor.

### Dendritic cell differentiation

Monocytes (1x10^6^/ml) were cultured in DC complete medium with 30 ng/ml IL-4 and 30 ng/ml GM-CSF in 96-well U-bottom plates for 5 days as described in our previous reports (34, 36) in the presence of 1:2 dilution of Tb CM, PgOMV Tb CM or without any conditioned media (control, -). On day 5, cells were harvested for flow cytometry analysis. Supernatants were collected and stored at -20 °C for subsequent ELISA. In some cases, monocyte-derived cells were also treated with LPS 200 ng/ml for 18 h. LPS stimulation was used to induce dendritic cell maturation and assess their functional responsiveness.

### Flow cytometry

Flow cytometry analysis was performed on monocyte-derived dendritic cells. Cells were washed with PBS containing 10% FBS and incubated with the following antibodies at saturating concentrations for 30 minutes at 4°C: FITC-conjugated anti-CD1a (Cat. 300103), PerCP-conjugated anti-CD14 (Cat. 325632), FITC-conjugated anti-CD83 (Cat. 305316), PE-conjugated anti-CD86 (Cat. 305405) and PE-conjugated anti-HLA-G (Cat. 335906), all from BioLegend, San Diego, CA, USA. Cells were then washed and fixed with 1% paraformaldehyde. In all cases, the stained cells were acquired using an FACSAria II cytometer (BD Biosciences) and results were analyzed using FlowJo^®^ version 10.8.1 Software. The gating strategy used for analysis is shown in **Supplementary Figure 1**.

### Measurement of cytokines by ELISA

Cytokine secretion was measured in supernatants collected from monocyte-derived DC cultures. IL-10 and TNF-α levels in DC supernatants were quantified using commercial ELISA kits (Cat. 555157 from BD Biosciences and Cat. 31673019 from Immunotools, respectively) according to the manufacturer’s instructions.

### Statistical analysis

GraphPad Prism 9 (GraphPad Software Inc., San Diego, CA, USA) was used to perform all statistical tests. Statistical significance was determined using parametric or nonparametric tests as appropriate. For parametric analyses, paired t-test was used. For nonparametric analyses, Wilcoxon test or Friedman test with Dunn’s multiple comparisons post-test were applied, as indicated in each figure legend. In all cases, the statistical significance was defined as p<0.05.

## Results

### Trophoblast cells primed with PgOMV enhance monocyte chemoattraction

**Figure 1:**
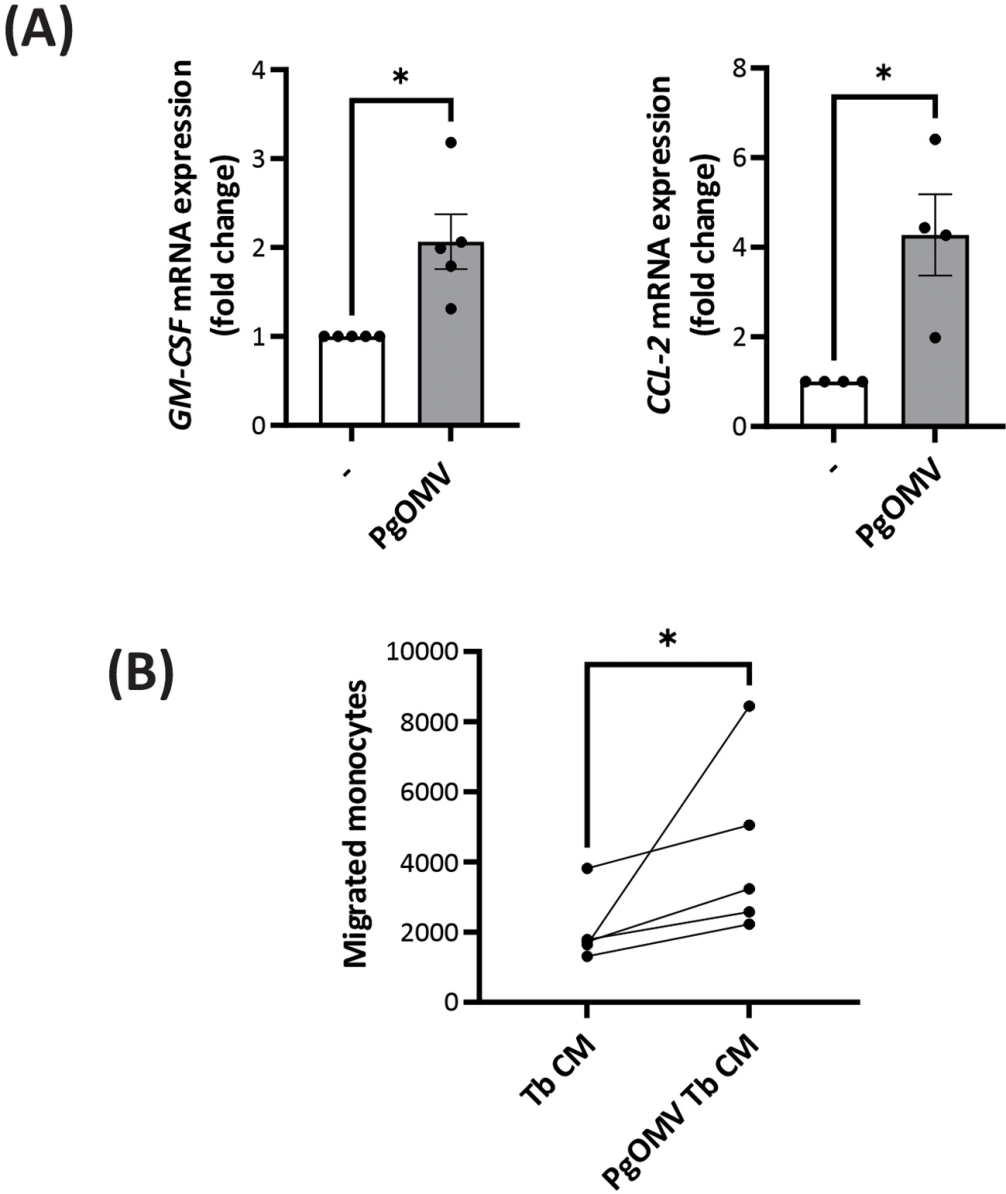
Conditioned media from PgOMV–treated trophoblast cells promotes monocyte migration. **(A)** Relative mRNA expression of GM-CSF (n=5) and CCL-2 (n=4) in HTR-8/SVneo cells cultured in the presence or absence of PgOMV (1 μg/ml) for 6h, as determined by qPCR. Data are expressed relative to control cells., *p < 0.05, Paired t-test. **(B)** Transwell migration assay of monocyte toward trophoblast conditioned medium (Tb CM), or conditioned medium from PgOMV–treated trophoblast cells (PgOMV Tb CM). Each dot represents an individual donor, and paired samples are connected. n=5, *p<0.05, Wilcoxon test.

Based on our previous findings reported (31), where PgOMV were shown to induce CCL-2 upregulation and neutrophil activation, we first evaluated whether extravillous trophoblast cells exposed to PgOMV exhibited changes in the expression of immune mediators involved in monocyte regulation. Given the well-established role of GM-CSF in promoting monocyte differentiation toward dendritic cells, we included this cytokine in our analysis. qPCR analysis revealed a significant increase in GM-CSF expression, together with the induction of CCL-2 (**Figure 1A**). Given the central role of these factors in monocyte recruitment, activation, and survival, we next asked whether conditioned media (CM) from OMV-treated trophoblast cells could promote monocyte migration. In this context, we found that PgOMV Tb CM significantly increased the number of migrated monocytes compared with Tb CM (**Figure 1B**).

### PgOMV reduce the potential of trophoblast cells to retain DC differentiation at a CD1a^-^CD14^+^ phenotype

**Figure 2:**
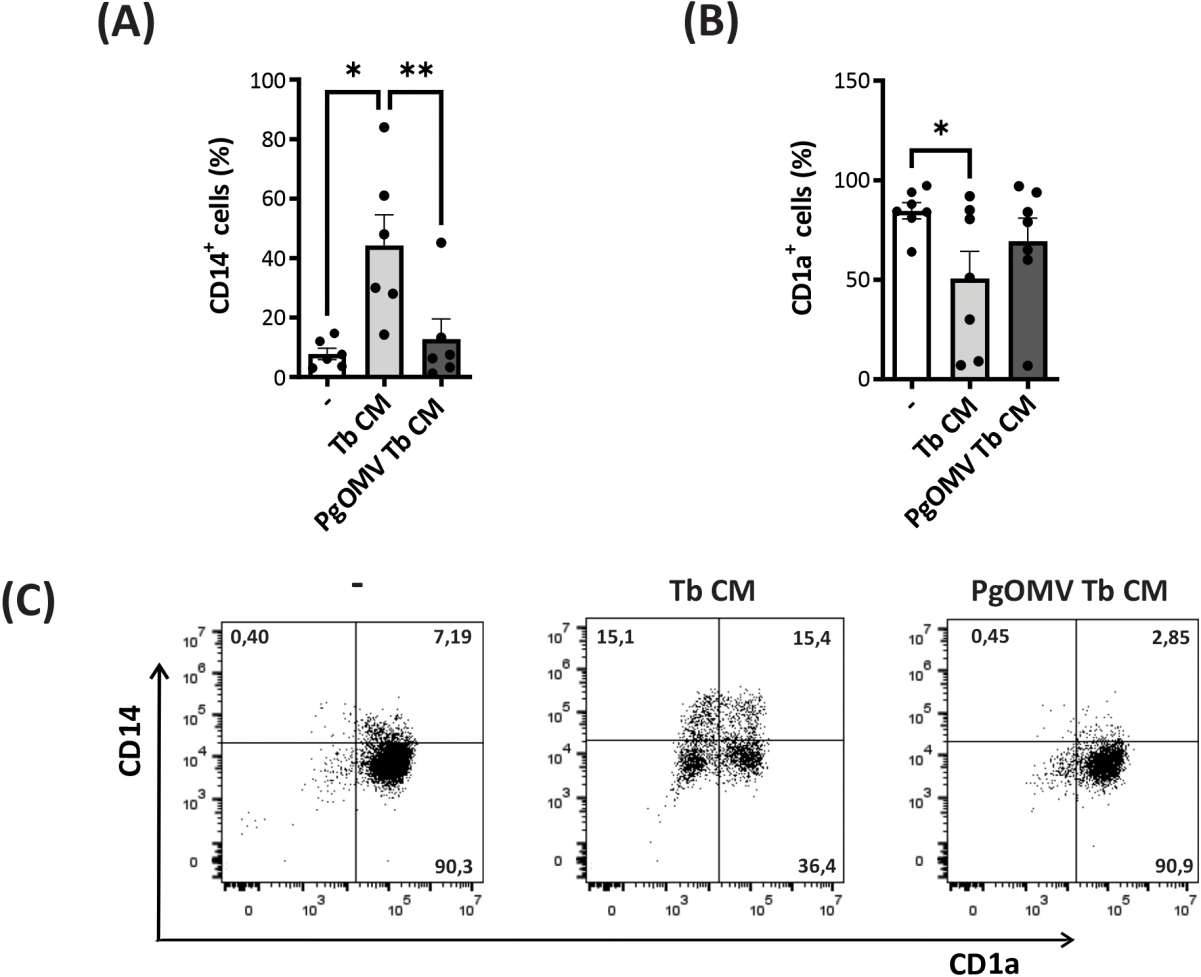
PgOMVs reduce the potential of trophoblast cells to retain DC differentiation at a CD1a^-^CD14^+^ phenotype. Monocytes were differentiated into DCs in the presence of rhGM-CSF + rhIL-4 alone (control, -) or supplemented with Tb CM or PgOMV Tb CM (1:2 dilution) for 5 days. **(A)** Frequency of CD14^+^ (n=5) and **(B)** CD1a^+^ (n=7) cells determined by flow cytometry. Each dot represents an individual donor. *p<0.05, **p<0.01; Friedman test with Dunn’s post-test. **(C)** Representative flow cytometry dot plots showing CD1a and CD14 expression under the three culture conditions.

Given that PgOMV impairs trophoblast-immune interactions by attenuating their anti-inflammatory effects on neutrophils (31), and considering that under physiological conditions, the trophoblast cells interfere with the acquisition of a classical immature DC profile by increasing the frequency of CD1a^-^CD14^+^ cells (20), we evaluated the impact of PgOMV priming of trophoblast cells in the conditioning of DC differentiation. Monocytes were cultured to differentiate into immature DC with rhGM-CSF + rhIL-4 in absence or presence of Tb CM or PgOMV Tb CM for 5 days. As expected, Tb CM inhibited monocyte differentiation into classical CD1a^+^ immature dendritic cells, as evidenced by reduced CD1a and increased CD14 expression, consistent with impaired monocyte-to-DC differentiation (**Figures 2A, B**). Notably, this effect was reversed when cells were differentiated in the presence of conditioned media from PgOMV-treated trophoblasts. **Figure 2C** shows representative dotplots of the immunostaining of DCs differentiated in the presence of different CM.

### PgOMV treatment on trophoblast cells prevents the conditioning of tolerogenic DC profile on monocyte-derived cells

Since CM from OMV-treated trophoblast cells altered the differentiation of DCs toward the classical immature profile, we analyzed their impact on the acquisition of a tolerogenic phenotype. To this end, the expression of the classical activation/maturation markers CD86 and CD83, respectively, as well as IL-10 and TNF-α secretion by ELISA, was evaluated.

**Figure 3:**
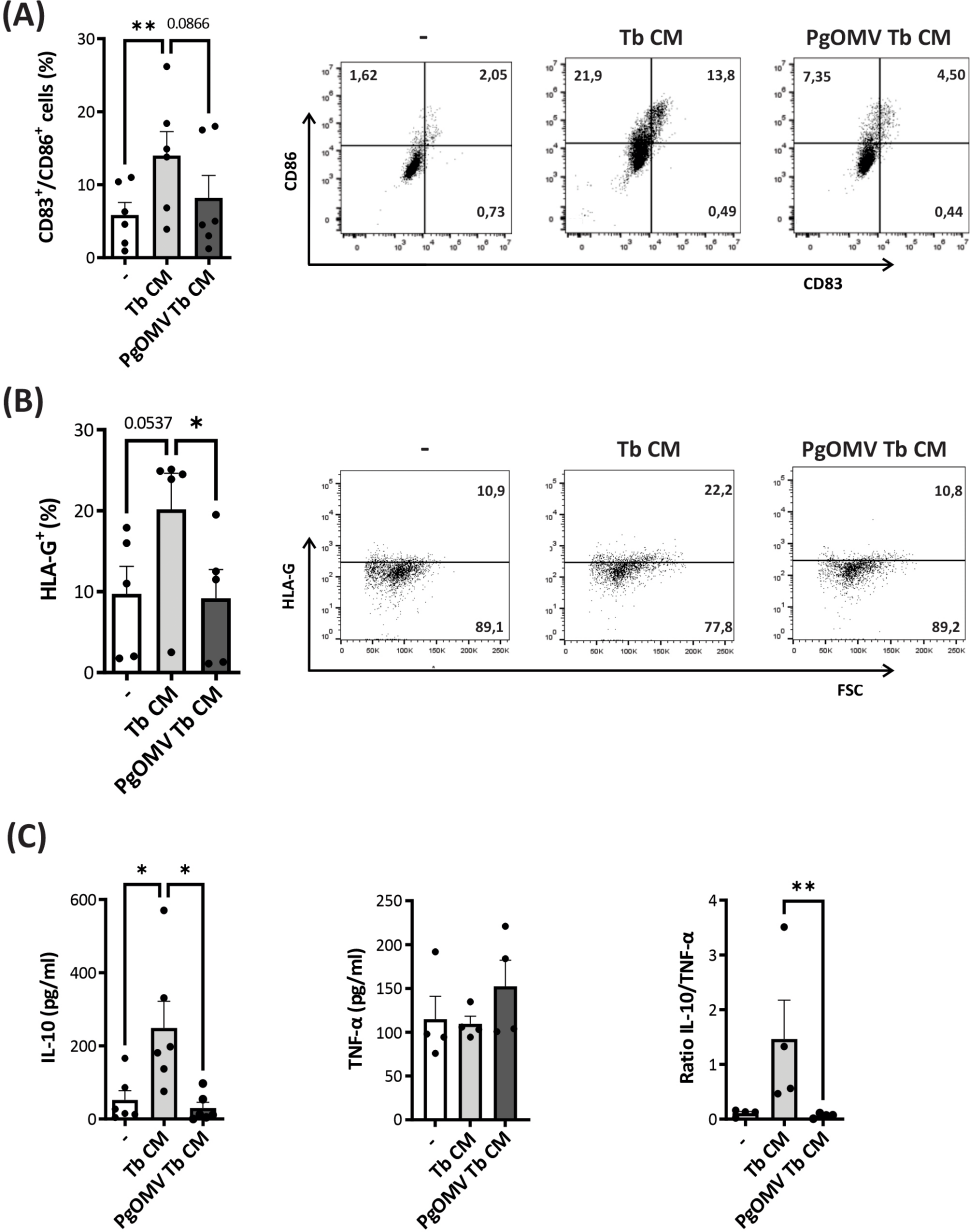
Trophoblast cells treated with PgOMV lose the ability to induce tolerogenic dendritic cells. Monocytes were differentiated into DCs in the presence of rhGM-CSF + rhIL-4 alone (control, -) or supplemented with Tb CM or PgOMV Tb CM (1:2 dilution) for 5 days. **(A)** Frequency of CD83^+^CD86^+^ cells determined by flow cytometry. Each dot represents an independent donor. Representative dot-plots are shown on the right. (n=6) **p<0.01; Friedman test with Dunn’s post-test. **(B)** Frequency of HLA-G^+^ cells (left) and representative experiment (right) are shown. Each dot represents an independent donor. (n=5) *p<0.05; Friedman test with Dunn’s post-test. **(C)** IL-10 and TNF-α secretion measured by ELISA in cell culture supernatants and IL-10/TNF-α ratio are shown. Each dot represents an independent donor. (n=6) *p<0.05, **p<0.01; Friedman test with Dunn’s post-test.

Monocytes differentiated in the presence of CM induced the generation of a population of CD83^+^CD86^low^ cells, associated with a mature but low-activated DC profile. Consistent with this observation, cultures differentiated in the presence of Tb CM showed significantly increased IL-10 secretion without changes in TNF-α levels, while HLA-G expression showed a marginal, non-significant increase (**Figures 3A–C**). In contrast, conditioned media from PgOMV-treated trophoblasts reshapes tolerogenic DC differentiation, as evidenced by a reduced frequency of CD83^+^CD86^low^ cells, decreased IL-10 production, and lower HLA-G expression compared with Tb CM. Consistently, the IL-10/TNF-α ratio was significantly diminished following PgOMV exposure, indicating a shift away from a regulatory cytokine balance. These findings suggest that PgOMV may alter trophoblast-derived soluble factors, thereby impairing their capacity to promote tolerogenic DC differentiation.

### PgOMV exposure abrogates the ability of trophoblast cells to attenuate LPS-induced DC maturation

**Figure 4:**
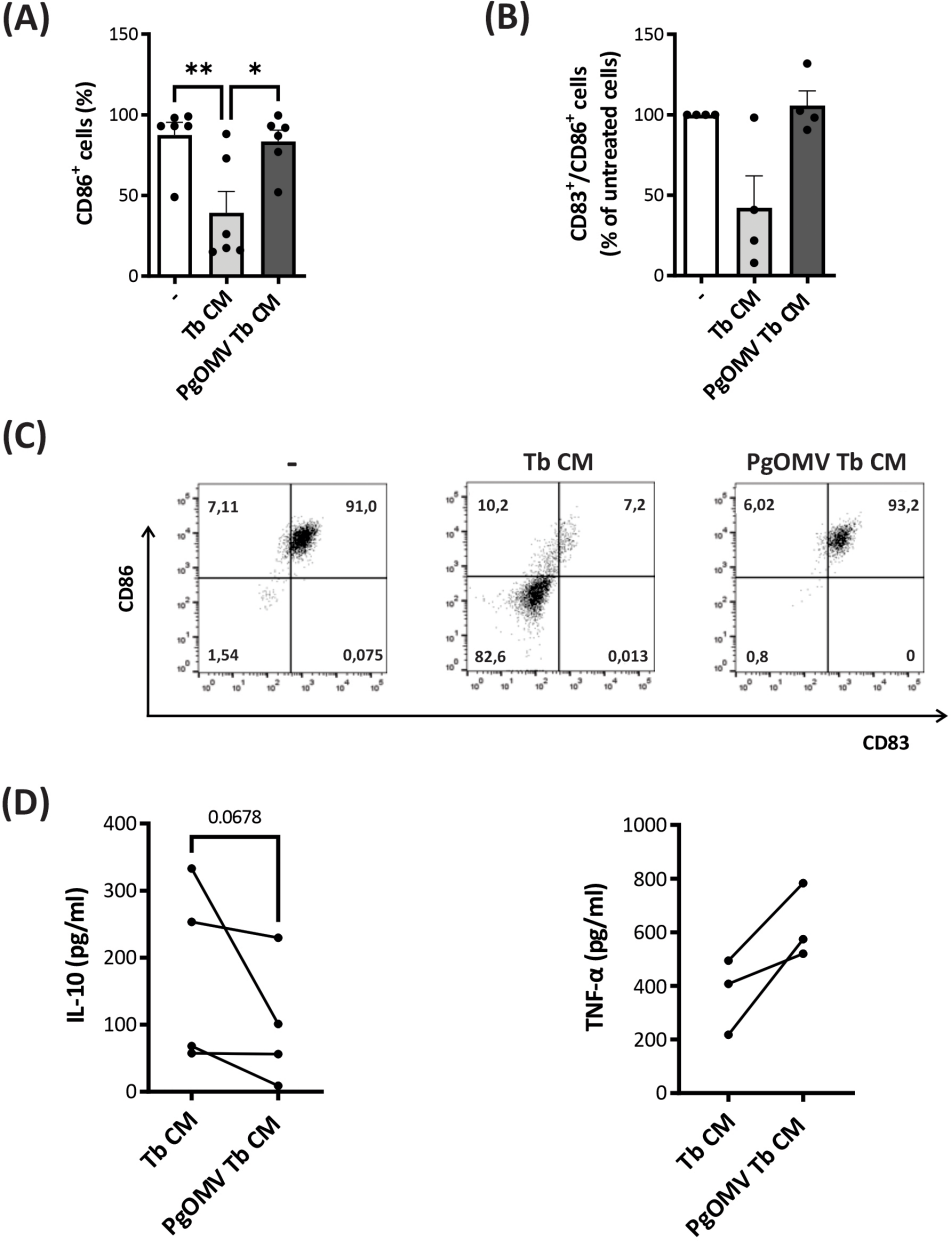
PgOMV exposure reverses the regulatory effects of Tb CM on DCs despite inflammatory stimulation. Monocytes were differentiated into DCs in the presence of rhGM-CSF + rhIL-4 alone (control, -) or supplemented with Tb CM or PgOMV Tb CM (1:2 dilution) for 5 days, and then stimulated with LPS (200 ng/ml) for 18h. **(A)** Frequency of CD86^+^ (n=6) and **(B)** CD83^+^CD86^+^ cells (n=5) was determined by flow cytometry after LPS stimulation. Each dot represents an independent donor. **(C)** Representative dot-plots are shown on the right. *p<0.05, **p<0.01; Friedman test with Dunn’s post-test. **(D)** IL-10 (n=4) and TNF-α (n=3) secretion measured by ELISA in cell culture supernatants. Each dot represents an independent donor. Wilcoxon test.

Finally, to determine the effect of PgOMV Tb CM on DC maturation and their ability to respond to pro-inflammatory stimuli, cells were challenged with LPS (200 ng/mL) for 18 h and the expression of CD83 and CD86 was evaluated. As expected, soluble factors released by trophoblast cells prevented the LPS-induced maturation of monocyte-derived cells, as evidenced by reduced expression in DCs activation/maturation markers (**Figure 4A**). On the contrary, when trophoblast cells were pre-treated with PgOMV, they lost this regulatory ability, restoring a classical LPS-driven mature DC profile comparable to control conditions (− or LPS alone), characterized by a higher frequency of CD86^+^ cells (**Figure 4A**) and an increased proportion of CD83^+^CD86^+^ cells (**Figures 4B, C**). Consistent with this profile, DCs differentiated in the presence of PgOMV Tb CM exhibited lower IL-10 and higher TNF-α secretion, although these differences did not reach statistical significance, supporting a shift toward a more pro-inflammatory state (**Figure 4D**).

Taken together, these results suggest that PgOMV exposure alters the immunomodulatory capacity of extravillous trophoblast cells, impairing their ability to promote a tolerogenic profile and allowing DC activation and maturation in response to inflammatory signals, which may reflect either the induction of pro-inflammatory mediators or a reduced production of tolerogenic factors.

## Discussion

In this study, we demonstrate that priming extravillous trophoblast cells with PgOMV disrupts their ability to condition monocyte-derived dendritic cells toward a tolerogenic phenotype, providing new mechanistic insights into how periodontal pathogens may compromise maternal-fetal immune tolerance during early pregnancy. The first trimester represents a critical window in which immune tolerance is actively established and is therefore particularly susceptible to inflammatory perturbations. Within this context, our findings reveal that PgOMV alters the secretory profile of first-trimester extravillous trophoblast cells, thereby preventing the establishment of regulatory DC phenotypes that are essential for pregnancy maintenance.

Under physiological conditions, trophoblast cells play a central role in shaping immune homeostasis at the maternal–fetal interface through the secretion of soluble factors that regulate the differentiation and function of local immune populations. In this regulatory network, DCs are key mediators of maternal–fetal tolerance, contributing to the control of local inflammatory responses and supporting placental development, including angiogenic processes, through coordinated interactions with trophoblast cells and other immune subsets (37–39).

Consistent with previous reports showing that trophoblast cells inhibit classical immature DC differentiation and promote regulatory CD14^+^ phenotypes (20, 29), we observed that conditioned media from untreated extravillous trophoblast cell line HTR-8/SVneo prevented the generation of CD1a^+^ DCs while maintaining a CD14^+^ cell population. Importantly, this regulatory effect was abrogated when trophoblast cells were pre-exposed to PgOMV, resulting in a restoration of the classical CD1a^+^ and CD14^+^ DC phenotypes. In addition, PgOMV-treated trophoblast cells lost their ability to induce key features of decidual tolerogenic DCs, including the CD83^+^CD86^low^ maturation state, increased IL-10 secretion, and HLA-G expression (28, 34, 40). Moreover, upon LPS stimulation, Tb-conditioned DCs exhibited resistance to inflammatory challenge by maintaining a stable, semi-mature, low-costimulatory phenotype with high IL-10 production—hallmarks of tolerogenic DCs—an effect that was abolished in PgOMVTbCM cultures. This result suggests that soluble factors released by PgOMV-treated trophoblasts may impair the tolerogenic conditioning normally induced by trophoblast cells, either through the induction of pro-inflammatory mediators or through a reduced production of tolerogenic factors, potentially favoring a more permissive environment for inflammatory responses during pregnancy. Notably, we have recently reported that trophoblast cells pre-exposed to PgOMV exhibit increased bacterial invasion and persistence (32), indicating that PgOMV exposure simultaneously compromises both immune tolerance and antimicrobial barrier functions at the maternal-fetal interface.

These findings position PgOMV as systemically disseminated, non-infectious bacterial effectors capable of remotely disrupting trophoblast-driven tolerogenic DC programming, thereby providing a biological basis for the association between periodontal disease and early pregnancy complications. Indeed, disruption of regulatory DC programs has been associated with early pregnancy complications such as recurrent pregnancy loss and preeclampsia (29, 41, 42), underscoring the importance of tightly controlled DC-mediated immune regulation during the first trimester. In line with this, we recently demonstrated that a stressed, pro-inflammatory DC profile negatively impacts trophoblast migration, resulting from impaired tolerogenic conditioning by stressed stromal cells (36), further highlighting the clinical relevance of altered DC programming at the maternal–fetal interface.

Collectively, these data indicate that PgOMV alter the trophoblast secretory profile thereby compromising its immunomodulatory capacity. In this regard, we observed that PgOMV exposure induced trophoblast cells to express higher mRNA levels of GM-CSF and CCL-2, resulting in enhanced monocyte migration. This is consistent with our previous findings showing that conditioned media from PgOMV-pretreated trophoblast cultures promotes neutrophil recruitment (31).

The role of GM-CSF is particularly relevant, as this cytokine not only promotes monocyte recruitment and survival but also critically influences their differentiation trajectory (43, 44). Although GM-CSF is required for conventional DC differentiation *in vitro* and, under inflammatory conditions, promotes the generation of DCs with enhanced immunogenicity, it can exert regulatory functions in specific immunological contexts. Indeed, GM-CSF has been reported to modulate DC differentiation toward tolerogenic, Treg-supportive phenotypes, depending on its concentration and the surrounding cytokine milieu. Accordingly, in pregnancy-related models, low-dose GM-CSF has been associated with enhanced tolerogenic DC traits (45, 46). However, excessive or dysregulated GM-CSF signaling may favor inflammatory DC differentiation. In line with this, pregnant women exhibit lower systemic GM-CSF levels and reduced frequencies of mature circulating DCs, consistent with a shift toward a less inflammatory, more tolerogenic DC compartment during gestation (47, 48). Thus, dysregulated GM-CSF production in the context of PgOMV exposure may contribute to a deviation from tolerogenic toward more inflammatory DC phenotypes at the maternal–fetal interface.

CCL-2 or Monocyte Chemoattractant Protein-1 (MCP-1), as its name implies, plays a central role in monocyte recruitment but also participates in shaping myeloid cell differentiation. In the first-trimester decidua, CCL-2 has been shown to modulate DC differentiation from CD14^+^ monocytes, favoring the maintenance of immature DC-SIGN^+^ DCs and limiting the emergence of mature CD83^+^ DCs. Nevertheless, other reports indicate that CCL-2 may also promote DC maturation, highlighting its context-dependent effects (49). Dysregulation of CCL-2 signaling may therefore disrupt the balance between immune tolerance and inflammation. Indeed, altered CCL-2 levels have been associated with adverse pregnancy outcomes, including spontaneous abortion, preeclampsia, and preterm labor, through effects on macrophage/DC composition, trophoblast invasion, and angiogenesis (49).

Although the coordinated increase in GM-CSF and CCL-2 expression in PgOMV-primed extravillous trophoblast cells, together with the enhanced monocyte recruitment and altered dendritic cell differentiation observed in our system, supports the potential involvement of these mediators, direct mechanistic evidence for their causal role is still lacking. Future studies employing functional blocking approaches, such as neutralizing antibodies against GM-CSF or CCL-2, will be required to determine the specific contribution of these factors to the immunomodulatory effects induced by PgOMV.

We acknowledge some limitations of the study especially in the interpretation and extension of our results to the clinics, since an experimental assay *in vitro* is used to model the maternal-placental interface. First, HTR-8/SVneo is an immortalized cell line that models extravillous trophoblasts but cannot fully recapitulate the complexity of the decidual microenvironment. Future studies using primary trophoblasts or freshly isolated decidual immune cells would help validate these findings in a more physiologically relevant context. Second, while the conditioned media approach allows the evaluation of soluble mediators released by trophoblast cells, it represents a simplified model that does not fully recapitulate the complexity of trophoblast–immune cell interactions at the maternal–fetal interface, where direct cell–cell contact may also contribute to immune regulation. In addition, although our results support the idea that trophoblast-derived factors mediate the observed effects on DC differentiation, the presence of small amounts of residual PgOMVs in conditioned media cannot be completely excluded. Third, although a total of nine independent donors were included across the study, not all donors contributed to every analysis, resulting in smaller sample sizes for specific experiments. This, combined with the inherent biological variability among donors characteristic of experiments using primary cells, may have limited the statistical power to detect significant differences in some analyses. Future studies with larger cohorts will be needed to confirm these trends. Also, it is important to acknowledge that direct evidence demonstrating the presence of PgOMV in gestational tissues in humans with periodontitis is currently limited. While it has been reported that OMVs from anaerobic bacteria can reach the placenta even during healthy pregnancies (50), suggesting a plausible route of dissemination, it remains unclear whether PgOMV in particular reach gestational tissues during human pregnancy and, if so, at what quantities. Our previous work in murine models, in which intraperitoneal OMV injection was used, demonstrated that PgOMV can reach gestational tissues (15), however this may not accurately reflect the systemic dissemination route during periodontitis in humans. Our findings should therefore be interpreted as providing mechanistic insight into a plausible pathway rather than direct *in vivo* evidence of OMV-driven immune dysregulation during pregnancy. Our *in vitro* approach nonetheless enables the dissection of highly specific mechanisms of trophoblast–immune crosstalk that would be extremely difficult to resolve in complex *in vivo* settings. In this regard, the explicit evaluation of OMV-driven interference in dendritic cell differentiation requires the use of highly purified monocyte populations and controlled differentiation systems, given the low frequency and phenotypic heterogeneity of first-trimester decidual DCs and the technical and ethical constraints associated with their isolation. Therefore, this model provides a necessary mechanistic framework to interpret how bacterial extracellular vesicles may disrupt immune tolerance pathways at the maternal–fetal interface.

While we identified GM-CSF and CCL-2 as altered mediators, the complete molecular signature induced by PgOMV exposure remains to be elucidated. Future studies employing proteomic and secretome-based approaches will be necessary to define the full repertoire of trophoblast-derived factors modulated by PgOMV, potentially revealing additional pathways and therapeutic targets. Moreover, dissecting the contribution of specific OMV components—such as gingipains, LPS, or bacterial nucleic acids—using purified virulence factors or genetically modified OMV would provide deeper mechanistic insight into pathogen-driven disruption of trophoblast–immune crosstalk at the maternal–fetal interface.

Together, these findings expand our understanding of how periodontal infection contributes to early placental immune dysregulation and identify, for the first time, a role for PgOMV in modulating trophoblast–dendritic cell crosstalk, a central axis in the establishment of maternal–fetal immune tolerance during early pregnancy.

## Data Availability

The raw data supporting the conclusions of this article will be made available by the authors, without undue reservation.
