# Supplementary material for: Porphyromonas gingivalis outer membrane vesicles prevent the induction of tolerogenic dendritic cells by first trimester trophoblast cells
Source: Front Immunol. 2026 May 8;17:1814664. doi: 10.3389/fimmu.2026.1814664 (PMC13194567; doi:10.3389/fimmu.2026.1814664)
Supplement: Supplementary file 1 [file Table1.docx]

***Porphyromonas gingivalis* outer membrane vesicles prevent the induction of tolerogenic dendritic cells by first trimester trophoblast cells**

Brenda Lara, Ana Schafir, Ailén Fretes, Lourdes Materazzi, Fátima Merech, Daiana Rios, Rosanna Ramhorst, Claudia Pérez Leirós, Daiana Vota, Soledad Gori†*, Vanesa Hauk†*


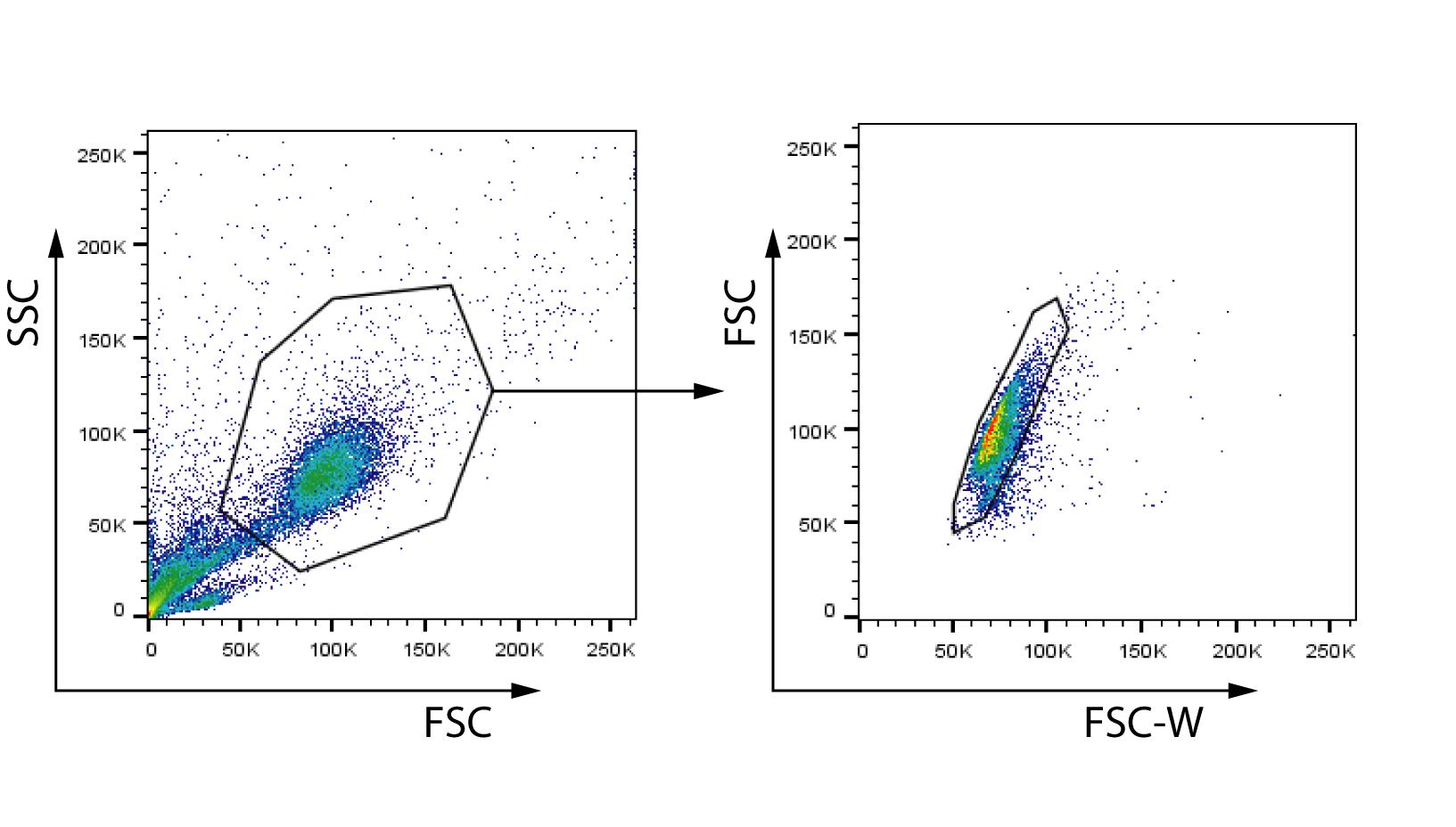


**Supplementary figure 1. Gating strategy**Cells were first gated based on forward scatter (FSC) and side scatter (SSC) parameters to exclude debris. Doublets were excluded by FSC-A versus FSC-W gating, and the resulting singlet population was used for further analysis. No viability dye was used. The expression of CD1a, CD14, CD83, CD86, and HLA-G was then evaluated within this population.
